# 893 The Relationship Between Serum Iron and Mortality and Length of Hospitalization in Burn Patients

**DOI:** 10.1093/jbcr/iraf019.424

**Published:** 2025-04-01

**Authors:** Zheyar Seyan, Merry Mathew, Taha Hassan, Babafela Awosile, Alan Pang

**Affiliations:** Texas Tech University Health Sciences Center School of Medicine; Texas Tech University Health Sciences Center School of Medicine; Texas Tech University Health Sciences Center School of Medicine; Texas Tech University Health Sciences Center School of Veterinary Medicine; Texas Tech University Health Sciences Center School of Medicine

## Abstract

**Introduction:**

The American Burn Association estimates that about 450,000 severe burn injuries requiring medical intervention occur annually. Blood loss and chronic inflammation from burns often lead to a state of anemia in patients. Iron levels are a significant biomarker in burn victims, yet the precise impact of iron on the recovery process in burn wounds remains unclear. The aim of this study is to investigate the relationship between serum iron concentration and mortality and length of hospitalization in burn patients.

**Methods:**

This retrospective study utilized data from patients admitted to a burn ICU between January 1, 2011, and August 1, 2022. The study aimed to investigate the relationship between serum iron levels and patient mortality and length of hospital stay. Multivariate regression analysis was conducted using data from 111 burn patients.

**Results:**

The analysis showed no significant difference in serum iron levels between patients who survived and those who succumbed to their injuries in the BICU. However, patients with serum iron levels ≥60 mcg/dL were less likely to die compared to those with < 60 mcg/dL (OR=0.21, 95% CI:0.01-64.15). There was no statistical association between serum iron level and length of hospitalization, although patients with serum iron level within or greater than normal range (≥60 mcg/dL) spent 11 days less compared to the patients with serum ion level less than 60 mcg/dl. We observed a statistically significant negative linear correlation between the length of hospitalization and serum iron level (Rho=-0.19, P =0.042).

**Conclusions:**

While this study did not find a significant difference in serum iron levels between survivors and non-survivors, it suggests that higher serum iron levels (≥60 mcg/dL) may be associated with lower mortality and shorter hospital stays in burn patients, indicating a potential benefit of iron supplementation. However, further research with a larger and more diverse patient population is needed to better understand the role of serum iron in burn care.

**Applicability of Research to Practice:**

This research provides valuable insights into the potential role of serum iron levels in burn patient outcomes, offering implications for clinical practice in burn care. Prior studies have identified an association between iron deficiency and increased mortality and poorer physical recovery in critical care patients. This study builds on the association between iron levels and clinical outcomes in burn patients. While this study found no statistically significant difference in serum iron levels between survivors and non-survivors, it suggests that higher serum iron levels (≥60 mcg/dL) may be associated with lower mortality rates and shorter hospital stays. This finding could inform treatment strategies, potentially supporting the use of iron supplementation in burn patients with low serum iron levels.

**Funding for the Study:**

N/A

